# Protein phosphatases at the nuclear envelope

**DOI:** 10.1042/BST20170139

**Published:** 2018-02-06

**Authors:** Raquel Sales Gil, Ines J. de Castro, Jerusalem Berihun, Paola Vagnarelli

**Affiliations:** 1 College of Health and Life Science, Research Institute for Environment Health and Society, Brunel University London, London UB8 3PH, U.K.; 2 Department of Infectious Diseases, Integrative Virology, University Hospital Heidelberg and German Center for Infection Research (DZIF), Heidelberg 69120, Germany

**Keywords:** chromatin, nuclear envelopes, nucleus, protein phosphatases

## Abstract

The nuclear envelope (NE) is a unique topological structure formed by lipid membranes (Inner and Outer Membrane: IM and OM) interrupted by open channels (Nuclear Pore complexes). Besides its well-established structural role in providing a physical separation between the genome and the cytoplasm and regulating the exchanges between the two cellular compartments, it has become quite evident in recent years that the NE also represents a hub for localized signal transduction. Mechanical, stress, or mitogen signals reach the nucleus and trigger the activation of several pathways, many effectors of which are processed at the NE. Therefore, the concept of the NE acting just as a barrier needs to be expanded to embrace all the dynamic processes that are indeed associated with it. In this context, dynamic protein association and turnover coupled to reversible post-translational modifications of NE components can provide important clues on how this integrated cellular machinery functions as a whole. Reversible protein phosphorylation is the most used mechanism to control protein dynamics and association in cells. Keys to the reversibility of the system are protein phosphatases and the regulation of their activity in space and time. As the NE is clearly becoming an interesting compartment for the control and transduction of several signalling pathways, in this review we will focus on the role of Protein Phosphatases at the NE since the significance of this class of proteins in this context has been little explored.

## Phosphatases in nuclear envelope reassembly

In higher eukaryotes, the nucleus is heavily remodelled during mitosis; this leads to the formation of mitotic chromosomes and the dissociation of the major components of the NE (nuclear envelope), the nuclear pore complexes (NPCs), and the nuclear lamina. This process requires the activation of several kinases including Cyclin-dependent kinase 1 (CDK1) and Polo-like kinase 1 (PLK1) [1,2], and the inactivation of protein phosphatases. Once the DNA is separated, the inactivation of CDK1 and PLK1 [2] coupled to the re-activation of protein phosphatases allows the original nuclear structures to be re-formed [3]. Since excellent reviews have dealt with the reformation of the nuclear envelope (NE) in mitosis [4–6], here we summarize only the contribution of phosphatases to the process.

Despite the numerous kinases involved in the Nuclear Envelope Breakdown (NEB) and the ever-growing mitotic phosphosites identified in several NE components by differential proteomic analyses, the number of phosphatases associated with specific NE reformation events is still very scarce. The first protein phosphatase shown to be involved in these events was protein phosphatase 1 (PP1) via its regulatory subunit AKAP149 [7]; this complex dephosphorylates lamin B during mitotic exit, promotes lamin B polymerization [8,9] and is required for lamina reassembly. Since this initial discovery, it took 14 years to unveil that another PP1 subunit, Repo-Man (also known as CDCA2), was responsible for binding and targeting Importin β to the chromatin periphery, thus facilitating NPC reassembly [10,11]. Repo-Man also interacts with Nup153 and its recruitment to the nuclear periphery is dependent on this interaction [12]. Nup153 is a heavily phosphorylated protein in mitosis and we could speculate that it could well be a Repo-Man substrate; however, direct evidence for this is still missing.

In *Caenorhabditis elegans* oocyte meiosis I, PP1 is recruited to the anaphase chromatin by MEL28/ELYS, an essential component for NE reassembly. This recruitment has been shown to be important both for promoting kinetochore disassembly and for ensuring the correct completion of nuclear compartmentalization [13]. The key substrates of this complex are yet not known.

At the same time, protein phosphatase 2A (PP2A) was shown to be essential for the de-phosphorylation of BAF (barrier-to-autointegration factor) [14], a protein that regulates chromatin structure, chromosome segregation, and gene expression, and that is essential for NE reformation in *C. elegans* [15] and Drosophila [16] as it interacts with LEM domain-carrying proteins (Lap2, Emerin, and Man1) of the INM and the lamina. At mitotic exit, the re-activation of PP2A counteracts VRK (vaccinia-related kinase) phosphorylation (which targets BAF in early mitosis [15] and enables BAF interaction with chromatin, LEM proteins and the lamina) thus contributing to the NE reformation [14].

Considering the number of NE components phosphorylated in mitosis that are targeted by multiple kinases, it is quite intuitive to predict that three phosphatases cannot suffice to explain the reassembly process. For example, out of the 32 phosphosites of Nup153 [17], only S257 and S1463 were recently shown to be dependent on PP2A/B55, thus arguing that other phosphatases are then required to complete the process.

Lamin A is also a major mitotic phospho-protein, but no phosphatases have yet been identified that are responsible for its de-phosphorylation. It has recently been shown that, at least for the S22 site, the interaction with a sumoylated chromatin-associated protein in anaphase is essential to remove the mitotic modification [18]. However, the nature of this phosphatase complex is still unknown.

The identification of the phosphatases involved in NE reassembly, together with the timing and localization of the de-phosphorylation events, will enable us to recapitulate and control the process in space and time.

## Phosphatases in interphase

The mitotic phosphorylation of NE proteins leads to its disassembly. However, many other phosphorylation events also occur in interphase, but their functions remain to be understood. In this section, we will explore what is known so far about phosphorylation of nuclear components beyond mitosis, their possible biological function, and how phosphatases could regulate these events.

### Reversible phosphorylation of nucleoporins in interphase

#### Regulation of import/export

The NPC is a ∼60 nm diameter gate (central channel) that serves to exchange molecules from the cytoplasm to the nucleus, and vice-versa. RanGTP (GTPase) and Importins (α/β) are the two main classes of proteins that bind cargos containing NLS (nuclear localization signals) and facilitate the trafficking process; small proteins can transit without these mediators. The Importin/cargo complex interacts with nucleoporins that provide the docking sites during the passage through the NPC.

Aside the well-documented effect of phosphorylation on specific cargos, particularly around the NLS domain, shown to enhance the binding to trafficking proteins (reviewed in [19]), phosphorylation of the NPC components can also contribute to a change in the cytoplasmic/nuclear permeability. The observation that phosphatase inhibition by Okadaic Acid (OA) impairs the import by Transportin and Importin β without affecting their cargo-binding capacity led to the hypothesis that phospho-switches regulate the import of proteins [20].

Although the phosphorylation of NUPS at the beginning of mitosis leads to the disassembly of this macromolecular structure [1,21], there is no evidence that phosphorylation of NPC components in interphase causes the partial disassembly of the complex, thus affecting this barrier. However, several NUPS are heavily post-transnationally modified. Specifically, half of the 30 different types of protein components in the NPC are glycosylated, particularly the ones containing multiple phenylalanine-glycine (FG) repeats. This glycosylation is specifically mediated by O-linked *N*-acetylglucosamine transferase, which attaches an *N*-acetylglucosamine (GlcNAc) moiety to Ser or Thr residues. These FG-Nups form a strict selectivity barrier with a high density of *O*-GlcNAc in the NPC to mediate bidirectional trafficking between the cytoplasm and nucleus. Although the role for *O*-GlcNAc in NPC structure and transport has been controversial, recent studies have shown that these modifications do play a role on the stability and permeability of the NPC. Mutations of known *O*-GlcNAc sites on Nup62 cause a decrease in its stability [22] and glycosylation of Nup98 makes the hydrogel more dynamic and enhances the entry of large NTR·cargo complexes [23]. An interesting aspect is the reversibility of the O-GlcNAc modification and its relationship with phosphorylation. Competition between O-GlcNAcylation and phosphorylation for occupancy of serine/threonine sites occurs by several distinct mechanisms and many proteins are reciprocally modified under different conditions at the same site by either *O*-GlcNAc or phosphate; this interplay can therefore be used by cells in different contexts to restrict or enhance the permeability of the nuclear barrier.

However, little is known about the kinases and phosphatases involved in the phospho-regulation of nuclear transport. ERK has been shown to target Nup50, Nup153, and TPR [24,25]. Interestingly, the phosphorylation of Nup50 reduces its affinity towards Importin β, suggesting that the transport of cargos bound by RanGTP and Importins could be defective upon Nup50 phosphorylation [26]; the partnership between Nup50–Nup153 also plays a role in the stability of Importin β and, consequently, transport [27]. ERK is a stress kinase and therefore is not surprising that some of the Nups phosphorylations occur during stress response. Nup50 phosphorylation is observed upon oxidative stress, via MEK (upstream of ERK) and PI3 signalling kinases [28]. However, ERK inhibitors have an effect in both stressed and non-stressed cells, up-regulating the transport. This suggests that ERK can potentially phosphorylate Nups at low levels even without stress induction [28,29]. In other cases, Angiotensin neuron stimulation activates MAP kinases that lead to the phosphorylation of Nup62. In this case, phosphorylated Nup62 helps in the trafficking of signalling molecules to the nuclei, namely STAT3, activating stress gene expression pathways [30]. In yeast, stress induces the phosphorylation of Nups for rapid transport of mRNAs from stressed response genes. Hog1 stress-activated protein kinase (SAPK) is the mastermind behind osmo-stress response, triggering the activation of several genes repositioned at the nuclear pores upon stress activation as shown by ChIP experiments [31]. This is a reminder of the gene gating theory postulated in 1985 [32] in which it was hypothesized that genes in close proximity to the pores would be more rapidly transported to the cytoplasm for processing. Indeed, this seems to be the case. Recently, it has been shown that Hog1 phosphorylates Nup1, Nup2, and Nup60 (human closest equivalent to Nup50 and Nup153 [31]), increasing the transport rate of these mRNAs to the cytoplasm.

Phosphorylation as a means to control nucleo-cytoplasmic trafficking seems to emerge as a conserved regulatory mechanism between species. It is tempting to speculate that controlling the transport and hence the availability of molecules inside the nuclei can have impact on gene expression, given the fact that transcription activators/repressors and other modulators of transcription are shuffled by these means.

Once transported through the pores, the presence of phosphatases on the other side could be important to facilitate the release of the cargo. However, the phosphatases conducting the opposite reaction are still not known, but could represent important targets for the regulation of these processes.

#### Import high-jacked: nucleoporin phosphorylation during viral infection

A common feature of retroviruses’ life cycle is the integration into the genome for the production of more viral particles and thus the passage through the NPC. In this context, it has been shown that Integrase or Capsid proteins, from the HIV-1 virus, establishes interactions with members of the NPC, namely Nup358 (RanBP2), Nup153, or TPR and that down-regulation of nucleoporins reduces viral infectivity (reviewed in [33]). In cardioviruses, single-strand RNA virus members of Picornaviruses, their Leader (L) protein binds RanGTPase (trafficking transporter) and induces phosphorylation of several nucleoporins, namely Nup62, Nup98, Nup153, and Nup214 [34–36]. The phosphorylations are potentially triggered by ERK/p38 kinases in an l-protein-dependent manner [35] and, the inhibition of phosphatases by OA increases the rate of Nups phosphorylation [36]. These phosphorylations of NPC components do not seem to have any impact on its assembly but rather on the functions as they block nucleo-cytoplasmic transport [37]. However, in other cases, like in some Herpes virus, viral mediated phosphorylation of Nups does lead to rearrangement of the NPC structure. For example, the Epstein–Barr viral kinase BGLF4 mediates the phosphorylation of Nup153 and, possibly indirectly, Nup62 leading to nuclear pore distension and re-distribution of these nucleoporins, as well as of SUN 1, SUN 2, and Emerin from the underlying inner NE, suggesting a more widespread impact on the NE structure.

In all these cases, the local counteracting phosphatases are not known, although it can be easily realized that a local inhibition/modulation of such a mechanism could be extremely beneficial in treating viral infections.

Adenoviruses seem to sequester PP2A to the nucleopore. The viral protein E4orf4 forms a complex with Nup205 and PP2A via its ARM domain, common to many viral proteins [38]. Although the function of these interactions is still not known, it has been suggested that Nup205 is present in a phosphorylated state in normal conditions and that the virus mediates its de-phosphorylation by recruiting PP2A.

The Nups most susceptible to phosphorylation in all these scenarios are the ones located at the outermost cytoplasmic or nucleoplasmic side of the NPC, suggesting that interactions with cargos and delivery in the nucleoplasm might be aided by Nups post-translational modifications to establish stronger interactions with signalling/viral molecules either to activate or block responses.

Taken together, these suggest that viruses have the potential of sequestering kinases/phosphatases at the NPC in order to promote viral infection by down-regulation of trafficking. On the other hand, it also brings phosphatases into the repertoire of potential targets for viral infections.

### Phosphatases at the nuclear periphery and chromatin regulation

#### DNA damage response

To avoid increased mutation rates and genomic instability, eukaryotic cells have evolved multiple mechanisms for rapidly repairing DNA lesions that occur during the cell cycle. However, not all DNA lesions are efficiently repaired and some are not repaired at all. Recently, several studies in budding yeast have shown that this persistent damaged DNA localizes to the nuclear periphery within 1–2 h of the damage in order to finalize the repair, and this localization is mediated by several NPCs, including Nup96, Nup133, Nup84, and Nup107, and some other nuclear proteins such as Heh2 and Mps3 [39–42]. Deletion of Mps3 N-terminal domain eliminates peripheral localization of persistent DNA lesions, indicating a central role of this protein in DNA lesions anchoring to the nuclear periphery [39]. However, Horigome et al. [42] identified differences in the DNA lesion interactions with nuclear pores and with the nuclear protein Mps3. While DNA lesion interactions with nuclear pores can occur at any point during the cell cycle, Msp3 binding is limited to S and G2 phases. Furthermore, the latter requires both the chromatin remodelling INO80 and the recombinase Rad51, whereas NPC interaction does not. These differences propose a distinct pathway for DNA repair at the two nuclear anchorage sites, although the molecular mechanisms are still elusive.

An essential step in the DNA damage response (DDR) is the activation of key components. In this respect, PP2A is essential for the activation of ATM, ATR, Chk1, Chk2, and p53 [43]. Moreover, many proteins involved in the DDR, including BRCA1, pRB, 53BP1, and Cdc25, harbour the RVxF PP1 binding motif [44,45] and are targets of PP1.

**Figure 1.**
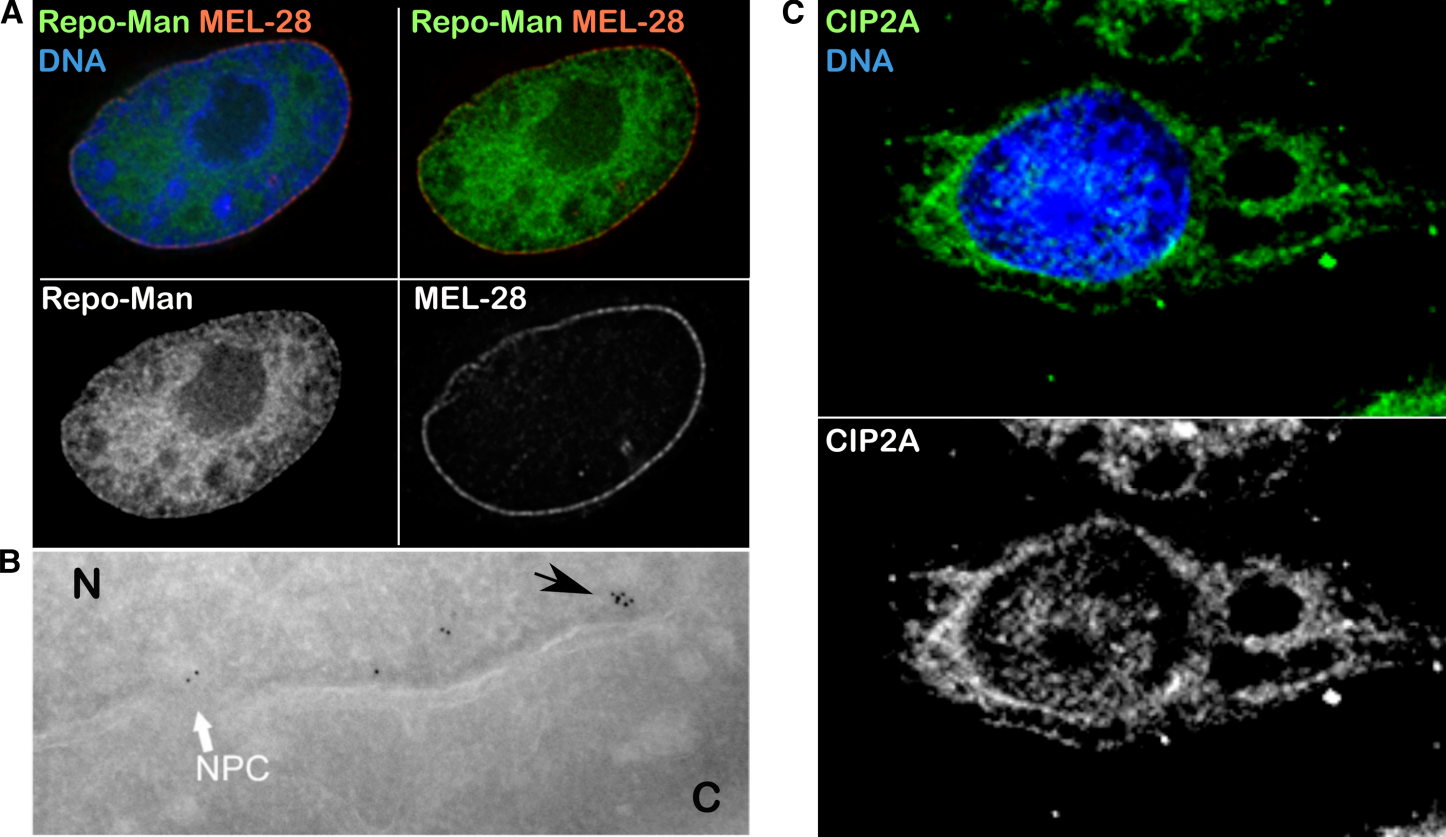
Localization of phosphatases at the nuclear periphery. (**A**) Localization of GFP:Repo-Man (green) and MEL-28 (red) at the nuclear periphery of HeLa cells. (**B**) Electron microscopy image of Repo-Man cell line expressing the peripheral N-terminus domain fused to GFP. Immuno-electron microscopy was conducted using an anti-GFP antibody. The white arrow shows accumulation at the edge of the NPC and the black arrow shows accumulation on heterochromatin adjacent to the NE. N, nuclear compartment; C, Cytoplasm. Image provided by Martin W. Goldberg, Durham University, UK. (**C**) Localization of CIP2A (green); picture kindly provided by Prof Jukka Westermarck, University of Turku, Finland.

Both PP1 and PP2A have been shown to localize at the nuclear periphery; PP2A via its inhibitor protein (CIP2A) [46] and PP1 via its chromatin targeting subunit Repo-Man [10] (Figure 1). Studies in *Xenopus* egg extracts demonstrated that Repo-Man interacts with ATM and PP1 through distinct domains, leading to PP1-dependent regulation of ATM phosphorylation and activation [47]. Following DNA damage, the Repo-Man/PP1γ complex is released from chromatin, leading to activation of ATM at DNA damage sites. If the accumulation of these phosphatases at the nuclear periphery has implications in specific aspects of DNA repair in mammals, it is an interesting hypothesis, but still needs to be demonstrated.

The relationship between phosphatases, DNA repair and NE becomes very important in the context of MYC regulation during intestinal crypt regeneration. MYC expression is essential for intestinal crypt regeneration in response to either irradiation-induced or chemically induced DNA damage [48,49]. Interestingly, MYC has been shown to associate with the NE [50,51], and MYC stability has been linked to its subnuclear partitioning [52]. Only recently, have the mechanisms behind these observations become clear. In fact, Myant et al. have shown that CIP2A promotes MYC S62Ph recruitment to the nuclear lamin A/C to maintain its activity. In this respect, CIP2A-Myc-lamin A interactions are critically linked to MYC capacity to reinitiate proliferation and support intestinal regeneration in response to DNA damage [46].

Together, these pieces of evidence, coupled also with recent mass spectrometry data [53], suggest the intriguing possibility of the existence of a regulatory multi-protein platform at the nuclear pores, including a protein phosphatase hub, that converge in the regulation of the DDR [54,55].

#### Regulation of nuclear-chromatin interactions

The nuclear lamina is a complex filament network that lines the NE and regulates nuclear size, shape, and mechanical properties. Besides these structural roles, lamins have also been shown to influence nuclear organization and gene expression by interacting with the chromatin [56]. These include interactions between nuclear lamins and core histones, Lamina Associated Polypeptide 2 (LAP2) and BAF, and interactions between lamin B receptor (LBR) and heterochromatin protein 1 (HP1) [57]. It is quite understandable that interactions with the lamina can vary between cells and during the cell cycle and that some dynamic aspects need to be locally regulated. In this context, numerous phosphorylation sites have been identified for lamin A in global, high-throughput, or large-scale proteomic studies [58,59], and it is easy to think that the phosphorylation status of the lamina will influence chromatin binding or its dynamics. These phosphorylations are important not only for the behaviour of lamin A during mitosis but also for the regulation of various aspects of the NE plasticity in interphase. Twenty interphase lamin A/C phosphorylation sites have been identified, of which almost half are high phosphate-turnover sites, and most of them are CDK or PKC targets [60]. Furthermore, the same S22 phosphorylation in lamin A that occurs in mitosis is also present in interphase albeit at a lower level. S22Ph is involved in the regulation of lamin A/C dimer head-to-tail polymerization and it has been shown that matrix stiffness coupled to myosin-II activity promotes lamin A/C Ser22 de-phosphorylation to regulate its turnover and physical properties [61]. Gajewski et al. [62] also demonstrate the requirement of LAP2α phosphorylation at four sites of the C-terminal domain for chromatin binding. This specific phosphorylation also generates a Pin1-binding motif in cells infected with human and animal alpha, beta, and gamma herpes viruses. Pin1 therefore represents a regulatory effector of lamina disassembly that could promote the nuclear pore-independent egress of herpes viral capsids via the regulation of PP2A activity.

In order to reverse these transient phosphorylation events, phosphatases need to act in a regulated manner, at the right place and at the right time. One of the phosphatases that play a role in organizing chromatin structure and tethering at the NE is Repo-Man. Our laboratory has recently shown that Repo-man is enriched at the nuclear periphery in interphase, where it is maintained via interaction with Nup153, and contributes to the reformation of HP1 foci after cell division by dephosphorylating H3S10ph and H3S28. De-phosphorylation of Repo-Man by PP1 and PP2A in anaphase allows Repo-Man binding to chromatin and is necessary for HP1 biding, the maintenance of a repressive chromatin environment, and localization of sub-telomeric regions at the nuclear periphery [12].

#### DNA replication

DNA replication (or firing) does not start simultaneously in all origins, but some origins replicate later than others. Several features are involved in the different timing of origin firing, including proximity to telomeres [63], transcriptional status, and nuclear positioning [64,65], where peripheral nuclear localization of chromosomal loci correlates with late replication in yeast and metazoan cells. Recently, Rif1 has been identified as a PP1-targeting subunit whose affinity is modulated by phosphorylation of its N-terminus by CDK1 and DDK [66]. Rif1 is enriched at the nuclear periphery during this phase of the cell cycle and co-localizes with late-replication origin [67]. In several organisms, Rif1-PP1 has been shown to be involved in repressing the activation of replication origins by limiting phosphorylation of Mcm2–7 complex [66,68–70] which could explain why late-replication origins are mostly localized at the nuclear periphery [71].

#### Conclusions and future perspectives

The NE is emerging as a cellular hub for numerous dynamic events during the cell cycle that allow the cell to grow, replicate, repair, and organize the genome. The phosphorylation status of the NE components regulates many of these processes.

**Figure 2.**
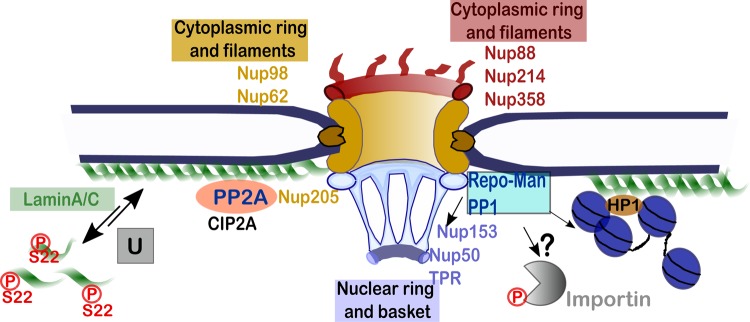
Phosphatases at the nuclear periphery. Repo-Man/PP1 regulates heterochromatin maintenance through histone H3 de-phosphorylation and interaction with Nup153. PP2A, PP1 (Repo-Man), and DNA damage proteins cluster at the nuclear periphery. Phosphorylation of laminA/C in interphase is linked to the regulation of NE stiffness: the phosphatase linked to this process is unknown (Grey box ‘U'). Repo-Man/PP1 binds Importin β and could represent a good candidate for the regulation of import/export. The nucleoporins (Nups) depicted are phosphorylated during viral infection (highlighted in the text).

Since alterations in many of these networks are linked to disease, the understanding of the key events will empower us with the knowledge necessary to investigate possible intervention strategies. While the role of kinases has been well studied, the counteracting phosphatases holo-complexes are still elusive and only a few have been so far identified (Figure 2).

However, if we search published proteomic datasets of NE preparations from different animal tissues, we can clearly identify the presence of a large number of phosphatases enriched in these fractions [72,73] (Table 1). These only represent the fraction tightly associated with the NE, but the identification of other phosphatases that are more enriched on the peripheral chromatin fraction (such as Repo-Man) will require a different preparation method and thus are still elusive. Interestingly, not all phosphatases are present in the same tissues, suggesting a tissue-dependent phosphatase activity.

**Table 1.** List of protein phosphatases enriched at the NE detected by MudPIT analyses of NE from Peripheral Blood Mononuclear Cells (PBMCs), liver, and muscle cells isolated from either rat, mouse, or human (from [72,73])

Gene	Predominant tissue
PPP1CA	Liver and PMBCs
PPP1CB	PMBCs
PPP1CC	Liver and PMBCs
PPP1R7	PMBCs
PPP1R8	Liver and PMBCs
PPP1R10	Liver
PPP1R12A	PMBCs
PPP1R16B	Liver
PPP2CA	PMBCs
PPP2R1A	Liver, muscle, PBMCs
PPP2R1B	PMBCs
PPP2R2A	PMBCs
PPP2R5C	Liver and PMBC
PPP2R5D	Liver
PPP2R4	PMBCs
SET	Liver, muscle, PBMCs
PPP4C	Liver
PPP6C	PMBCs
PPM1F	PMBCs
INPP1	PMBCs
INPP4A	PMBCs
PPM2C	PMBCs
ILKAP	Liver and PMBCs
ENPP4	PMBCs

Identifying the proteins involved in the phospho-switches of NE components would be a crucial step to understand how this cellular machinery behaves and could potentially identify possible targets to treat pathologies not only directly related with the NE but also with cell division, chromatin organization, and viral infections.

## Abbreviations

BAF: barrier-to-autointegration factor
CDK1: Cyclin-dependent kinase 1
DDR: DNA damage response
HP1: heterochromatin protein 1
LAP2: Lamina Associated Polypeptide 2
LBR: lamin B receptor
NE: nuclear envelope
NLS: nuclear localization signals
NPCs: nuclear pore complexes
OA: Okadaic acid
PBMCs: peripheral blood mononuclear cells
PLK1: Polo-like kinase 1
PP1: protein phosphatase 1
PP2A: protein phosphatase 2A
SAPK: stress-activated protein kinase
